# Water-Soluble Photoinitiators in Biomedical Applications

**DOI:** 10.3390/polym12051073

**Published:** 2020-05-07

**Authors:** Wiktoria Tomal, Joanna Ortyl

**Affiliations:** 1 Faculty of Chemical Engineering and Technology, Krakow University of Technology, Warszawska 24, 31-155 Krakow, Poland; wiktoria.tomal@doktorant.pk.edu.pl; 2 Photo HiTech Ltd., Bobrzyńskiego 14, 30-348 Krakow, Poland

**Keywords:** water-soluble photoinitiators, type I photoinitiators, type II photoinitiators, two-photon initiators (2PP), photopolymerization, biomedical applications, free-radical photopolymerization, cationic photopolymerization

## Abstract

Light-initiated polymerization processes are currently an important tool in various industrial fields. The advancement of technology has resulted in the use of photopolymerization in various biomedical applications, such as the production of 3D hydrogel structures, the encapsulation of cells, and in drug delivery systems. The use of photopolymerization processes requires an appropriate initiating system that, in biomedical applications, must meet additional criteria such as high water solubility, non-toxicity to cells, and compatibility with visible low-power light sources. This article is a literature review on those compounds that act as photoinitiators of photopolymerization processes in biomedical applications. The division of initiators according to the method of photoinitiation was described and the related mechanisms were discussed. Examples from each group of photoinitiators are presented, and their benefits, limitations, and applications are outlined.

## 1. Introduction

**Figure 1:**
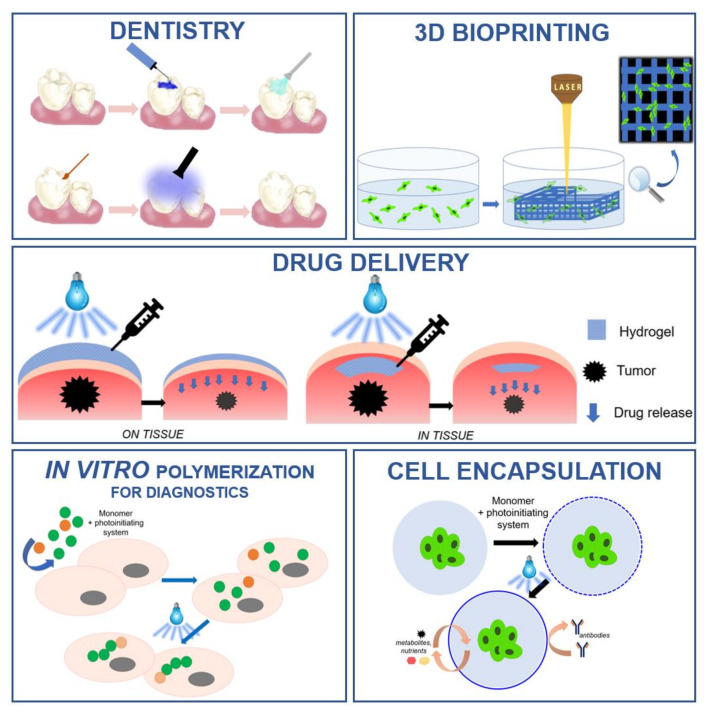
Examples of light-induced polymerization processes in biomedical applications.

Currently, polymerization processes are one of the most widely used chemical processes in various fields of industry [1,2]. One of the most modern and rapidly developing methods of obtaining polymers is light-induced polymerization, i.e., photopolymerization [3,4,5,6]. The technique of converting liquid monomers to solid polymers under the influence of applied light is widely developed in the polymer materials sector in the industry of solvent-free paints [7], varnishes [8], and adhesives [9], in optoelectronics [10], in the printing industry for 3D printing materials [11,12,13,14,15,16,17], and many others. Numerous advantages of photopolymerization, such as performing reactions at ambient temperature, lack of solvents, and extremely short processing times, made light-initiated polymerization perfectly suited for biomedical applications (Figure 1) [18,19]. 

The global market for photopolymerization in biomedical applications can be divided into various groups based on the area of application in the medical sector. The main segments are: dentistry [20,21,22,23], tissue engineering [24,25,26,27,28,29], bioimaging [30,31], drug delivery systems [32,33,34,35], and medical devices. In dentistry, photochemical-initiated processes are used for the filling of hard dental tissue cavities with photocured polymer composites [36,37,38,39]. An interesting application of photopolymerization processes is the production of photo-crosslinked polymeric biomaterials especially those based on totally or partially degradable materials [40,41,42,43,44], scaffolds for tissue culture [45,46,47,48,49], and diagnostic genetic or cellular matrixes [50,51,52,53,54,55,56,57,58].

The unquestionable advantages of the photopolymerization technique in the context of applications in tissue engineering and biomedical science are primarily its ability to form structures of any geometry as well as the deposition of such materials on various carriers. Lack of these possibilities is often a limitation of the functionality of biomaterials obtained through conventional polymerization processes.

Due to the mechanisms of polymerization as well as the type of used monomers and initiating systems, there is a distinction between radical photopolymerization and cationic photopolymerization, which are the basic processes used in light-initiated polymerization technologies. Radical photopolymerization is a chain reaction consisting of three main stages: initiation, propagation, chain growth, and termination (which may be accompanied by side reactions) [59]. Free-radical photopolymerization is mainly used for acrylate and methacrylate monomers. The factor that limits the usefulness of radical photopolymerization is the occurrence of oxygen inhibition caused by the presence of atmospheric oxygen during the polymerization process. The negative influence of oxygen on polymerization is reflected, for example, by extinguishing the excited states of the initiator, which, in turn, affects the efficiency of the whole process. It is the free-radical polymerization, however, that is mostly used in biomedical applications, as proven by numerous literature reports [60,61,62,63,64]. 

The second type of polymerization is cationic photopolymerization, which is particularly interesting and relatively widespread in industrial applications, since it has a number of major advantages that make this process practical [65]. The living nature of cationic photopolymerization guarantees that the reaction continues to be effective even after shutting down the radiation source [66]. This enables a high degree of conversion to be achieved, which plays an extremely important role in the industrial practice. For this reason, photoinitiated cationic polymerization is becoming increasingly prevalent in global markets as an easy and energy-saving method for obtaining cross-linked polymers [67,68]. Despite its numerous advantages, cationic polymerization is very unlikely to be used in biomedical applications. One of the reasons is that cationic initiators generate strong protonic acids during initiation, whose acidic character negatively affects cell cultures [69]. The second reason is the sensitivity of cationic photopolymerization to moisture and water. Numerous scientific articles prove that the presence of water slows down or inhibits the polymerization reaction [70]. In addition, water can act as a chain transfer agent and promote the growth of new chains, which reduces the average molecular weight of the obtained polymer [71].

One of the basic requirements of photocuring systems used in biomedical sciences is their total or partial solubility in water. Water-based photocuring systems have already garnered interest since the late 1970s. Even then, it was well known that the use of water as a non-toxic, green, and cheap solvent was the solution to many problems related to the classical, organic compositions [72]. In addition, aqueous formulations can, in many cases, provide a reaction efficiency that cannot be achieved with conventional organic systems. Interestingly, the oxygen concentration in aqueous systems is an inch lower than in organic preparations, which significantly reduces oxygen inhibition for radical photopolymerization processes. Therefore, the use of water-soluble photoinitiators in aqueous systems for light-initiated polymerization is of great importance in the rapidly growing medical industry, and this article provides an overview of the literature related to the development of water-soluble initiators and their use in biomedical applications.

## 2. The Dynamics of the Development of Water-Soluble Photoinitiators

**Figure 2:**
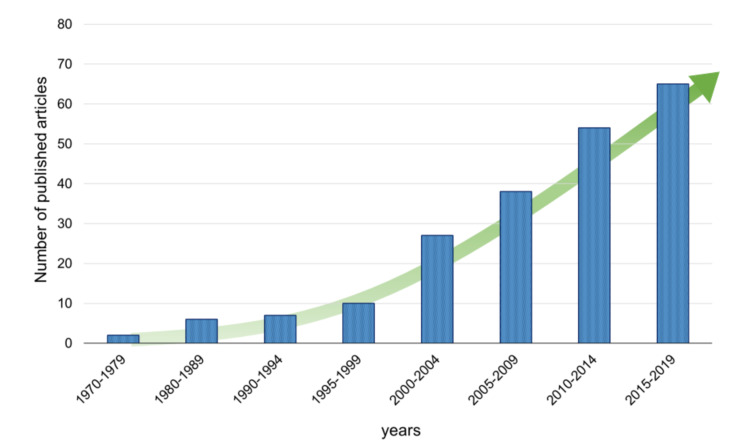
Number of published articles in the years 1970–2019.

The key role in light-initiated polymerization processes is played by the initiating system, which influence, among others, the speed of polymerization and the degree of monomer conversion. This has led scientists to concentrate on the development of photoinitiators, which poses major challenges. The dynamics of developing water-soluble photoinitiators have been visualised in Figure 2 as the number of articles published in the analysed subject matter between 1970 and 2019.

As can be seen, photoinitiators have their origins in the 1970s, when waterborne compositions gained popularity in the painting and coating industries, but the increase in water solubility did not always follow the required lack of toxicity of these initiators. A genuine breakthrough was made at the beginning of the 21st century when popular initiators, such as 2-hydroxy-1-[4-(2-hydroxyethoxy) phenyl]-2-methyl-1-propanone (Irgacure 2595) and water-soluble derivatives of acylphosphine oxides, e.g., monoacylphosphine oxide (MAPO) and bisacylphosphine oxide (BAPO), were used in new biomedical applications and started to play an important market role. The development of these initiators resulted in an increasing interest in the use of photoinitiated polymerization processes in biomedical applications, which reflects the high level of published scientific articles. Since then, scientists have worked on improving water solubility, initiation efficiency, and cytotoxicity reduction of the initiators discovered in the year 2000. Innovative photoinitiators designed depending on their application, e.g., initiators for two-photon laser polymerization, have also been proposed. An overview of the articles relevant to this topic can be found in the following chapters.

## 3. Types of Photoinitiators for Photopolymerization Processes

**Figure 3:**
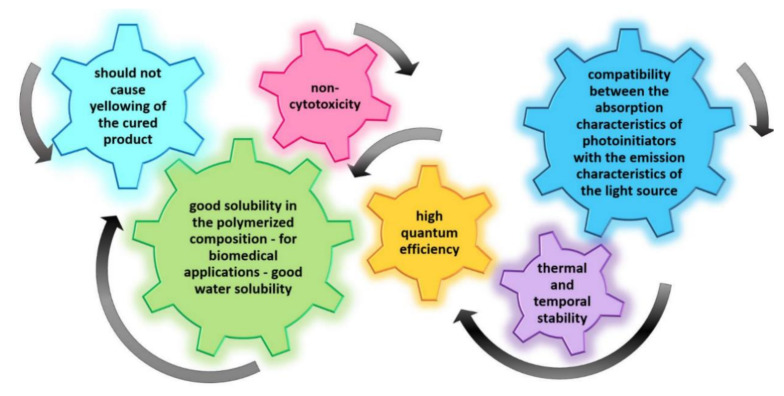
Highlights of initiators’ requirements.

The initiating systems based on one-component, two-component, or multi-component photoinitiators undoubtedly play a key role in photopolymerization processes [73,74,75,76,77]. Photoinitiating systems not only determine the mechanism of the reaction, but also affect its performance, curing speed, and final properties of the polymer, such as hardness and viscosity. The selection of a photoinitiator is essential to achieve the right photopolymerization reaction rate and the desired polymer properties. The basic parameters determining the selection of the photoinitiator are maximum absorption wavelength λ_max_ and a molar extinction coefficient ε. The efficiency of the photoinitiator is directly related to its structure, which influences the range of absorption and quantum efficiency of the photochemical and photophysical processes taking place in excited states [78]. Regardless of the type and mechanism of initiation, the photoinitiator should exhibit the following features (Figure 3):

- compatibility between the absorption characteristics of photoinitiators and the emission characteristics of the light source,
- high quantum efficiency,
- good solubility in the polymerized composition – for biomedical applications – and good water solubility,
- non-cytotoxicity,
- should not cause yellowing of the cured product, and
- thermal and temporal stability.

Other factors to be taken into account when performing the photopolymerization reaction are the structure and physicochemical properties of the monomers, the phenomenon of oxygen inhibition (in the case of free-radical polymerization), the influence of stabilisers or other additives present in the monomers, the thickness of the polymerizing layer, the type and intensity of the light source, and the viscosity of the composition.

In the case of an in vivo photopolymerization reaction, it is particularly important to reduce the toxicity of the initiator, especially when exposed to light. Free radicals produced during initiation may react with the main components of living cells, such as proteins and nucleic acids, which may affect the condition and viability of cells. Based on the mechanism of initiation of photoinitiators, a distinction is made between radical and cationic photoinitiators. In biomedical applications, radical photopolymerization processes are dominant.

**Figure 4:**
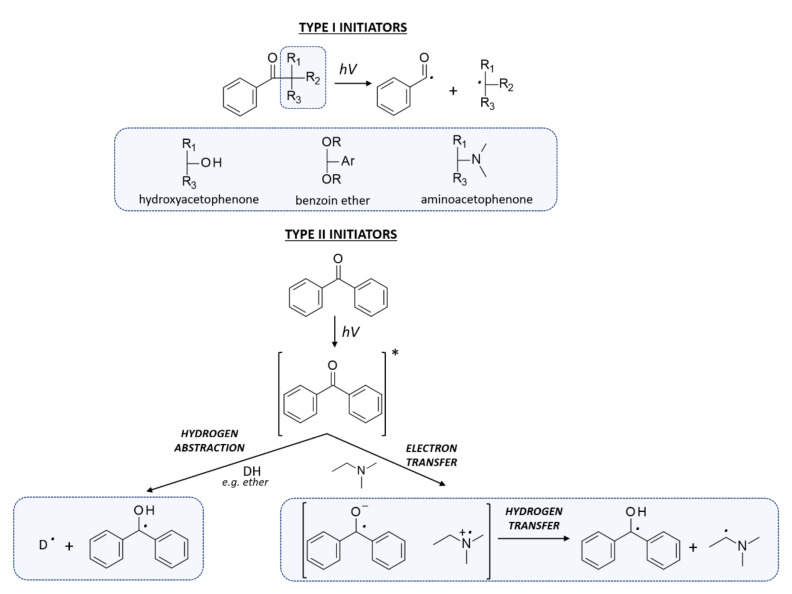
The photo initiation process using: A. type I initiator, B. type II initiator.

Free-radical photopolymerization is an example of a classic photochemical chain reaction in three main stages: initiation, propagation, and termination, which leads to the formation of oligomers or polymers [79,80]. Depending on the structure of a radical photoinitiator, free radicals may be formed in the process of homolytic photodissociation of the photoinitiator molecule – type I photoinitiators. This group of photoinitiators includes peroxides, peresters, iminosulphones, or ketones, where photofragmentation is performed by binding, for example, O-O, S-S, S-N or C-C at α or β – carbon atom to the carbonyl group [69]. In the case of Type II photoinitiators, the excited initiator molecule reacts with the appropriate co-initiator such as an electron donor or acceptor or a hydrogen donor in order to produce the appropriate radicals or radical-ions [81]. The photo initiation process using type I or type II initiators is presented in Figure 4. Types I and II photo initiations are single- and two-molecular processes, respectively. The second type is usually slower and less efficient due to the presence of competitive processes during the excitation of the photoinitiator by the monomer, co-initiator, and atmospheric oxygen. Conversely, the photon energy in the visible range is generally lower than the dissociation energy of individual organic compound bonds. Therefore, it is particularly difficult to obtain a highly efficient initiator operating in the visible range. Because of that, it is often in this range that the bimolecular systems are used.

**Figure 5:**
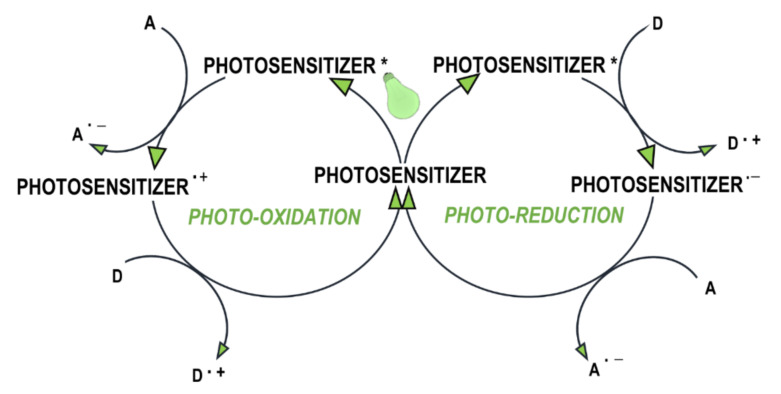
Initiation in multi-component systems: D – electron donor, A – electron acceptor.

Currently, multi-component photoinitiation systems, based on electron transfer, and systems based on hydrogen abstraction, are interesting options. The reaction of electron transfer is based on the interaction of an excited electron donor or acceptor with a second component (electron acceptor or donor respectively) in the ground state, which is responsible for the photoinduced electron transfer process. An excited photosensitiser molecule, as the primary light absorber in multiradical systems, can perform a dual role (Figure 5) [82]:

- where the photosensitiser acts as an electron donor, the transfer of the electron to the co-initiator creates a cationic radical of the sensitizer particle and an anionic radical of the co-initiator;
- where the photosensitiser is an electron acceptor, it undergoes photoreduction, and the electron transfer products are the anionic radical formed on the sensitizer molecule and the cationic radical formed on the co-initiator.

**Figure 6:**
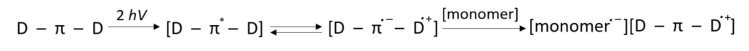
Schematic mechanism of initiation using two-photon photoinitiators.

In addition to the classic single, binary, and multi-component photoinitiators, there are also two-photon initiators (2PP) that undergo two-photon polymerization. This type of process is a powerful tool to build a variety of 3D matrices with micro-accuracy and nano-accuracy. A two-photon polymerization process is characterised by high penetration depth and high spatial selectivity. In this case, it is possible to use live cells to create 3D structures, thanks to the use of low-energy photons, which are safe for cells [83,84]. Two-photon photoinitiators should be sensitive to absorption because, during the initiation, they absorb two photons from the near infrared (NIR) area. In addition, they are characterised by highly conjugated π-systems and strong donor–acceptor groups [85]. The initiation process is not fully clarified, but it is suspected that, after absorbing the photons, the electron is transferred from the initiator’s donor–acceptor group to the π-electron core [86]. The transfer of the electron between the initiator and the monomer generates an exciplex and results in the formation of radicals that initiate the polymerization reaction (Figure 6) [87].

## 4. Type I Initiating System for Free-Radical Photopolymerization

### 4.1. α-hydroxyketones and Their Derivatives

**Figure 7:**
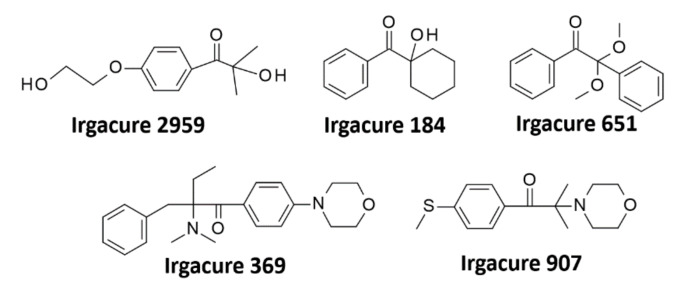
Various initiators from the Irgacure family used in biomedical applications.

One of the basic methods for increasing the solubility of traditional radical photoinitiators is their chemical modification, which consists of adding appropriate groups to the structure of the photoinitiator [88] [89,90,91]. The groups designated for this purpose are: non-ionic ethers, polyethers, hydroxyethers [92], ionic substitutes such as quaternary ammonium salts, sulphonates, carboxylic acids, and thiosulphates [93,94,95]. The most common solubilising group is the hydroxyl group, which can be found in the most popular water-soluble initiator: Irgacure 2959 – 2-hydroxy-1-[4-(2-hydroxyethoxy) phenyl]-2-methyl-1-propanone. This initiator contains ketone groups as functional groups and it is one of the first commercially available water-soluble photoinitiators to be used in a variety of areas. Despite its drawbacks, such as low water solubility below 2% and a narrow absorption range reaching only the UV-A − 365 nm range, this initiator has become widespread and a range of water-soluble initiators has been created on its core. One of the most important advantages of this group of initiators is the possibility of inexpensively modifying the primary carboxylic group [96,97,98]. One of the disadvantages of Irgacure 2959, as well as its derivatives, is the need to use UV light. Its maximum absorption is at 276 nm and, due to its poor absorption, Irgacure 2959 requires extended exposure time. As well known, the use of such a light source for cross-linking processes in biomedical applications has a significant negative impact on the functioning of cells, which causes their mutation or death [99,100,101]. Other derivatives from the Irgacure family have also been tested for biological purposes, including Irgacure 184 (1-hydroxy-cyclohexyl-phenylketone), Irgacure 369 (2-Benzyl-2-dimethylamino-1-(4-morpholinophenyl)-1-butanone) [102], and Irgacure 907 (2-Methyl-4′-(methylthio)-2-morpholinopropiophenone) [103,104,105]. Williams et al. compared the cytotoxicity of Irgacure 2959, Irgacure 651 (2,2-dimethoxy-2-phenylacetophenone), and Irgacure 184 [106]. The following relationship has been noted for all initiators. Initiator toxicity grows with increasing concentration of the initiator as well as with increasing exposure time to UV light. Irgacure 2959 turned out to be the best of this group, as the remaining two proved to be toxic to cells at a minimum concentration. The structures of α-hydroxyketones and their derivatives are shown in Figure 7.

**Figure 8:**
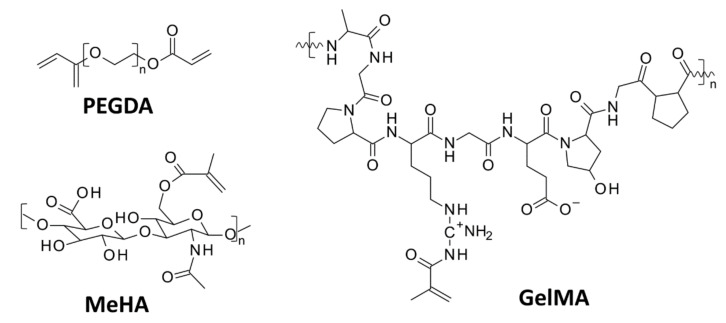
Commonly used monomers for preparing hydrogel materials by a photoinitiated polymerization reaction.

Irgacure 2959 is a type I photoinitiator that, when irradiated, cleaves into two radicals, benzoyl and alkyl, which can both initiate a polymerization reaction [107,108]. Irgacure 2959 is a widely used photoinitiator for preparing hydrogel materials using poly(ethylene glycol) diacrylate – PEGDA [109,110,111,112,113], gelatin-methacryloyl – GelMA [114,115], and methacrylated hyaluronic acid – MeHA [115,116] (Figure 8). This initiator is also used for cell encapsulation [117,118,119,120,121], for the targeted delivery of drugs and cells [122,123], and for the production of scaffolds for cell cultures [124,125,126,127,128].

Liska et al. have developed new water-soluble initiators containing carbohydrate residues and co-polymerising derivatives of these residues. The noted water-soluble initiators consisted of alkylphenones, benzophenones, and thioxanthones, and were accompanied by carbohydrates such as glucose and cellulose [129]. The proposed initiators proved to be highly effective in the initiation process, and those based on known structures, e.g., Irgacure 2959, have great potential in biomedical applications.

Another group of initiators, proposed in 1998 by Kojim et al., are based on 2-benzyl-2-(dimethylamino)-1-(4-morpholinophenyl)-1-butanone (BDMB), and more specifically on its water-soluble derivative: sodium 4-[2-(4-morpholino)benzoyl-2-dimethylamino] butylbenzenesulphone (MBS) [130]. Over time, the popularity of the MBS initiator and its modifications led it to find its way into biomedical applications, e.g., the microfabrication of scaffolds [131,132] and the printing of protein microstructures [133].

### 4.2. Phosphine Derivatives

**Figure 9:**
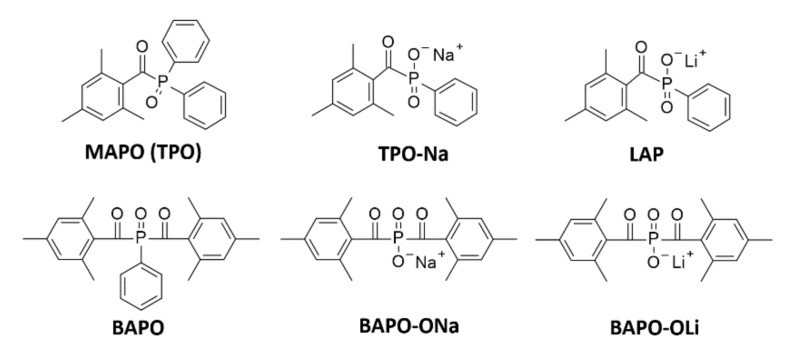
Monoacylphosphine oxide (MAPO) and bisacylphosphine oxide (BAPO) water-soluble derivatives.

Currently, scientists are working on modifying the already known initiators in order to either increase their water solubility or increase their absorption range and, consequently, obtain a fast and efficient initiating system [134]. Mono-acylphosphine oxides (MAPO) and bi-sacylphosphine oxides (BAPO) are mainly water-insoluble initiators that absorb in the 380–450 nm range. One of the first commercially available mono-acylphosphine initiators is TPO – diphenyl(2,4,6-trimethylbenzoyl)phosphine oxide (Figure 9). This initiator absorbs in the range of 350–380 nm. During initiation, it decays into reactive radicals, which provide high efficiency in the polymerization process. Its advantages also include good thermal stability and lack of colour and odour. This initiator, however, is poorly soluble in an aqueous medium [135].

Therefore, TPO derivatives with increased water solubility were created. The first reports of water-soluble initiators being TPO derivatives date back to 1991, when Majima et al. synthesised lithium phenyl-2,4,6-trimethylbenzoylphosphinate LAP, which proved to have good spectroscopic properties and high water solubility [136]. Their work was continued by Fairbanks et al. who, in 2009, improved the synthesis of the LAP initiator [137]. LAP is a widely used initiator for obtaining hydrogel materials using: PEGDA [138], GelMA [139], and other monomers [140,141].

Benedikt et al. analysed various modifications of bisacylphosphine oxides and compared the spectroscopic characteristics, polymerization kinetics, and cytotoxicity of the following derivatives: BAPO-OLi and BAPO-ONa (Figure 9) [142,143]. Both modifications were suitable as highly effective initiators for obtaining hydrogel materials. Additionally, BAPO-OLi ensured high cell viability [144]. The basic spectroscopic properties of MAPO-based and BAPO-based initiators, as well as the comparison of their solubility and toxicity, are presented in Table 1. Wang et al. performed a modification of BAPO by grafting its structure into a polyethylene glycol (PEG) chain, which improved its water solubility and allowed it to print a hydrogel with high optical resolution and good mechanical parameters [145]. 

In addition, scientists Pawar et al. have developed TPO water-dispersible nanoparticles, characterised by an absorption range of 380–420 nm, while maintaining a high molar excitation coefficient and good solubility in water [146]. TPO nanoparticles were prepared by rapid conversion of volatile microemulsions into water dispersible powder. This is a process that can be applied to various photoinitiators. Neither chemical modification of TPO nor the addition of organic solvents were required to obtain an efficient initiator, which maintains the outstanding spectroscopic properties of the TPO nano initiator and provides efficient 3D printing of hydrogel materials such as the production of highly stretchable hydrogels using digital light processing (DLP) [147].

### 4.3. Azo-Initiators

The water-soluble azo-initiator – 2,2’-azobis[2-methyl-N-(2-hydroxyethyl) promionamide] (VA-086) – becomes increasingly popular because of its low cytotoxicity in both precursor and radical forms, while its absorbance range offers the possibility of using different sources in the far UV range [148]. Occhetta et al., in their research on the production of hydrogel microstructures, have proven that the use of the VA-086 initiator allows for a very high optical resolution printout and also provides a high cell viability rate even after long exposure to light. Occhetta’s 3D micro-pellets were not only biocompatible, but also created an environment favourable to proliferation [149].

In turn, Wang et al. proved that the VA-086 initiator has great potential in tissue engineering, where the light source is a laser diode. By appropriately selecting the diameter of the beam and its burning time at one point, the final degree of conversion of the obtained polymeric materials can be controlled [150]. They also proved that the problem of obtaining hydrogel porous materials, caused by nitrogen release during the initiation reaction with the VA-086 initiator (Table 2), can be solved by appropriate exposure time selection. Han et al. proposed a two-component initiating system combining the initiator Irgacure 2959 and VA-086, which resulted in improved mechanical properties of the obtained polymer network, with a minimised radiation dose and reduced exposure time [151].

## 5. Type II Initiating System for Free-Radical Photopolymerization

### 5.1. Eosin-Y

**Figure 10:**
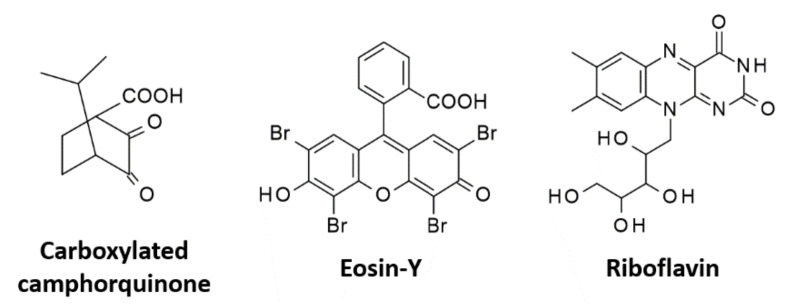
Type II initiators used in biomedical applications.

Eosin-Y is used as a photoinitiator due to its excellent spectroscopic properties, which makes it suitable for use with light sources in the visible range and safe for living organisms. Eosin-Y is an example of the type II initiator, which needs a second molecule, such as an electron donor, to initiate a polymerization reaction [152] (Figure 10). An example of such a co-initiator, which will be reduced during the reaction, is amine, e.g., triethanolamine. After the absorption of light, eosin is excited to a triplet state and then becomes an acceptor of the electron given by the amine. As a result of this process, eosin’s radical anion and radical cation of the amine are formed. Then, as a result of proton transfer from the amine radical cation, two neutral radicals are formed: the amino radical and the eosin radical (Table 2) [153,154]. Work on the Type II initiating system, Eosin-Y with amine, started in 1991 by Fouassier, Sawhney, et al., during which hydrogels were produced based on polyethylene glycol (PEG) [155]. As an initiating system, Eosin-Y/ethylamine was used in ratios of 0.4% w/v and 3.5% w/v, respectively [156]. Eosin has become very popular in the process of surface polymerization for the encapsulation of living cells, including islets of Langerhans [157,158,159]. Another important application of Eosine-Y is targeted drug delivery [160]. Eosin-Y is a widely used initiator for obtaining hydrogel materials, using mainly poly(ethylene glycol) diacrylate [161,162] and gelatin-methacryloyl [163]. In addition, Shih et al. have proven that Eosin can successfully act as a single-component photo initiating system in a thiol-ene photo-click polymerization reaction [164].

### 5.2. Riboflavin (B_2_)

Riboflavin is a naturally occurring yellow pigment, which is widely used in biomedical applications due to its high water solubility and biocompatibility [165] (Figure 10). Thus, the use of riboflavin as an initiator for hydrogel production would not only be harmless to cells but even beneficial. Riboflavin’s spectroscopic characteristics are favourable. It has a wide absorption range with four maximums: 223 nm, 267 nm, 373 nm, and 444 nm [166], and absorbs strongly between 330 and 470 nm, which makes it particularly attractive as an alternative to other synthetic initiators [167].

Riboflavin is a type II photoinitiator, which requires the presence of a co-initiator as an electron donor during the initiation of the polymerization reaction. Therefore, various initiating systems were studied. Bertolotti et al. examined a riboflavin/triethylamine initiation system for the photopolymerization of methacrylate hydrogels, which proved to be very efficient [168,169,170,171]. In order to produce a hydrogel free of unnecessary chemicals, however, the scientists proposed to use L-arginine as a co-initiator in the initiation process with riboflavin, since it contains amino groups as amino acids, which are electron donors [172]. Furthermore, in addition to initiating polymerization processes effectively, this co-initiator is biocompatible and well soluble in water. Additionally, it has been proven that the small concentration of riboflavin (0.01–0.5 wt.%) provides the fastest cross-linking as well as good physicochemical properties for the obtained hydrogel, while using 10% amine as a co-initiator [165]. The generation of riboflavin radicals and, thus, the reaction rate is also strongly influenced by the applied pH. In addition, it has been proven that following irradiation with visible light and UV light in the presence of oxygen, riboflavin produces reactive oxygen species such as: singlet oxygen, peroxide anion radicals, and others [173,174]. As a photoinitiator, riboflavin has already been used in in vivo studies for the treatment of corneal-related diseases, and the resulting hydrogels have proven to have promising physicochemical properties [175].

### 5.3. Camphorquinone and Its Modifications

One of the most popular initiators is camphorquinone (CQ), which belongs to the aliphatic α-ketones (Figure 10). The efficiency of this initiator in a one-component system is insufficient, while adding a second component, e.g., in the form of a tertiary amine as an electron donor, increases the efficiency of initiation. The mechanism is based on the process of electron–proton transfer [176]. Such combinations are widely used for the cross-linking of tooth fillings based on methacrylate resins [177]. Unfortunately, as an initiator in the visible range, camphorquinone has its drawbacks. First of all, it gives a strongly yellow product after the polymerization reaction, which makes the end product aesthetically unappealing. Additionally, camphorquinone has poor solubility in water, which limits the possibility of using this initiator to create hydrogel polymer networks [178].

In order to increase the water solubility of camphorquinone, it was modified to obtain carboxylated camphorquinone, while maintaining good spectroscopic properties [179,180]. The most commonly used co-initiators with CQ are amines: triethylenamine and ethyl-4-*N,N*-dimethylaminobenzoate (Table 2) [181,182,183]. Ternary initiation systems were also studied, which proved to be very effective and harmless to cells: the initiator, camphorquinone, the co-initiator, amine, and the accelerator, thioxantone or iodine salt [104,184]. The main direction of application of this initiator is the production of hydrogels for targeted drug delivery and in situ polymerization [185,186,187,188,189] as well as the production of biodegradable hydrogels for tissue engineering or biocompatible materials for application, e.g., in temporomandibular joints [190,191].

## 6. Two-Photon Photoinitiators (2PP) for Free-Radical Photopolymerizations in Biomedical Applications

**Figure 11:**
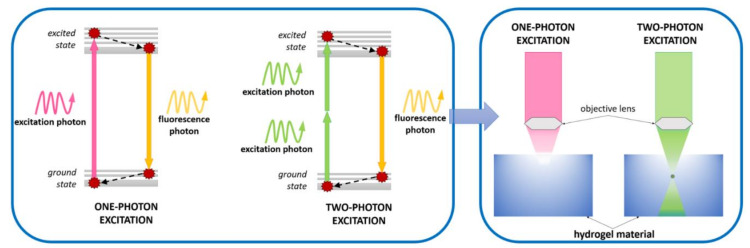
Comparison of one-photon and two-photon polymerizations.

As mentioned earlier, two-photon polymerization is a powerful tool for building 3D matrices with accuracy even on a nanometric scale, and, at the same time, enables spatial control and high depth of light beam penetration [192]. The light source is used in a near-infrared (NIR) region, which makes it possible to conduct the process in the presence of living cells [193]. The use of two-photon technology has, therefore, attracted significant interest in recent years [194,195,196,197,198,199]. For this technique, it is essential to select a suitable two-photon initiator which, when used in biomedical applications, must be water soluble, thermally stable, optically stable in the dark, non-toxic, and should generate free radicals easily [200,201]. The comparison of one-photon and two-photon polymerization is shown in Figure 11.

**Figure 12:**
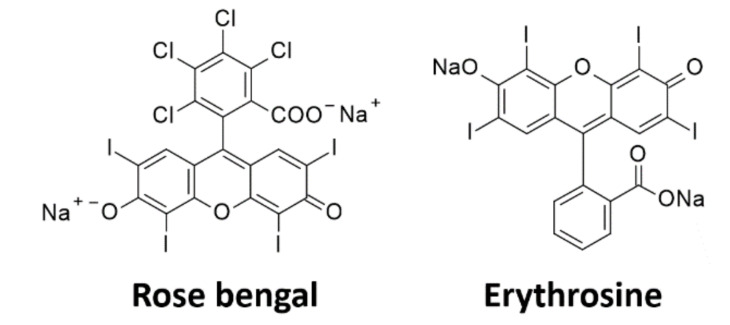
Dyes used in the two-photon polymerization process.

The challenge for two-photon initiators is to increase their water solubility. One of the methods for increasing hydrophilicity is the addition of non-ionic surfactants. Jkaverii et al. carried out two-photon polymerization using a commercially available initiator from the Irgacure family, Irgacure 651, with the addition of a surfactant, AF240 [202]. The disadvantage of this solution is the unfavourable influence of surface agents on the biocompatibility of obtained materials [203]. Initiators that undergo homolytic decay during irradiation, such as LAP [204] or VA-098 presented earlier, are characterised by relatively low π-system conjugation, poor two-photon absorption, and are, thus, less efficient in processes using laser as an irradiation source [205]. The popular Irgacure 2959 initiator can also be used in this case [206], but it is only suitable for 2PP at 515 nm [207,208]. Chichkov et al. used Irgacure 369 as a 2PP initiator in order to obtain biocompatible scaffolds. This compound, however, has a small cross-section in the NIR range [209] and its absorption maximum is at 369 nm, which makes it inconvenient when using a laser light source in the 750–800 nm range [210,211,212]. Some well-known type II initiators, such as Eosin-Y [213], erythrosine [214], and rose Bengal [215,216,217,218,219], in combination with amines, can be used as two-photon initiators of polymerization (Figure 12). The long exposure time and high intensity of radiation, however, makes it necessary to create initiators that ensure fast cross-linking using a small amount of light. Tromayer et al. proposed the preparation of a two-photon macromolecular initiator, which is based on cyclic dibenzylidene ketones and hyaluronic acid as the initiator core [220]. A biocompatible 2PP type initiator was obtained, which ensured the efficient initiation of the two-photon polymerization reaction and high cell viability, while hyaluronic acid, as the initiator’s core, provided it with adequate solubility in an aqueous medium.

**Figure 13:**
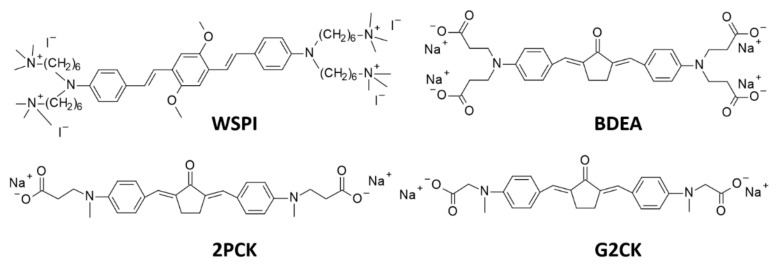
Examples of two-photon initiators used in biomedical applications.

The most effective way to increase water solubility is to introduce functional groups, such as quaternary ammonium salts or carboxylic salts, into the chromophore core, which is known for its high two-photon activity. Woo et al. have introduced quaternary ammonium cations for this purpose and the resulting WSPI initiator (1,4-bis(4-(*N,N*-bis(6-(*N,N,N*-trimethylammonium)hexyl)amino)-styryl)-2,5-dimethoxybenzene tetraiodide) has led to the creation of hydrogel materials containing living cells [221,222]. WSPI was also used to obtain protein hydrogels based on thiol and vinyl copolymers [223]. Another group of two-photon initiators includes compounds for which water solubility is guaranteed by the incorporation of carboxylic groups into the structure. This group includes BSEA (2,5-bis-[4-(diethylamino)-benzylidene]-cyclopentanone [224]), P2CK (3,3’-((((1E,1’E)-(2-oxocyclopentane-1,3-diylidene)bis(methanylylidene))bis(4,1-phenylene))bis(methylazanediyl))dipropanoate) [225,226], and G2CK (sodium 2,2’-((((1E,1’E)-(5-methyl-2-oxocyclohexane-1,3-diylidene)bis(methanylylidene))bis(4,1-phenylene))bis(methylazanediyl))diacetate) (Figure 13).

**Figure 14:**
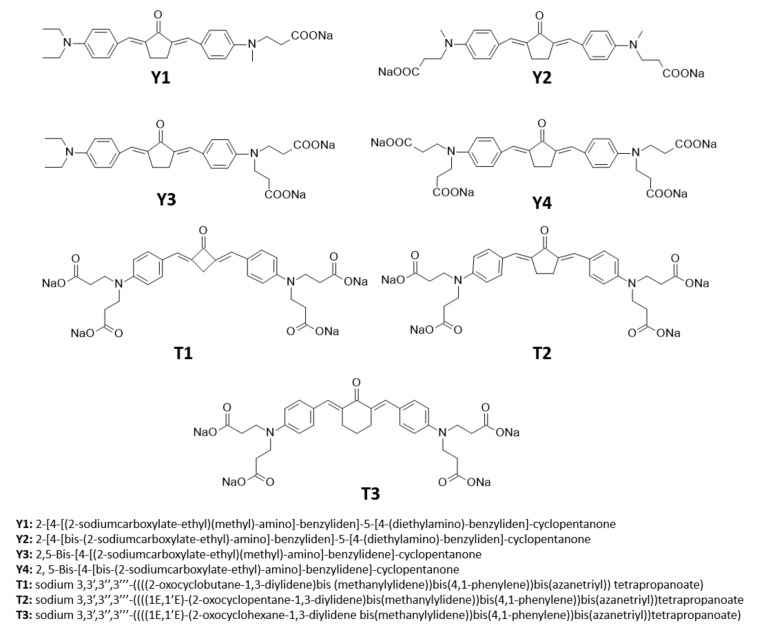
Water-soluble, new initiators containing sodium salt of propionic acid.

Currently, a promising application of two-photon absorption is two-photon excited photodynamic therapy (TPE-PDT), which, due to its deep penetration, lack of cytotoxicity, and high selectivity, is widely studied and developed. The selection of appropriate photosensitisers is key to effective TPE-PDT. Such a compound must meet the following criteria: hydrophilicity, biocompatibility, and non-toxicity to cells. In order to increase water solubility, the structure of two-photon initiators should be modified, such as by introducing carboxylic groups or attaching polyethylene glycol particles to the chain. Yang et al. proposed a series of carboxylate modified benzylidene cyclopentanone (Y1–Y4) as potential sensitizers for use in two-photon therapy [227]. The structures and full names of the initiators are shown in Figure 14. All obtained derivatives were characterised by a broad range of absorption in the visible range, and studies, including EPR, proved that the proposed water-soluble compound can effectively generate radicals. It was shown that the introduction of a larger number of hydrophilic groups into the initiator’s structure increases the biological safety of this initiator due to the lower probability of such a molecule being captured by cells [228]. Huang et al. proposed a new series of initiators (T1–T3), which not only initiated the two-photon photopolymerization process effectively but also ensured high cell viability [229]. The structures and full names of the initiators are shown in Figure 14.

## 7. Inclusion Complexes of the Host-Guest Type: Photoinitiator—Cyclodextrin

**Figure 15:**
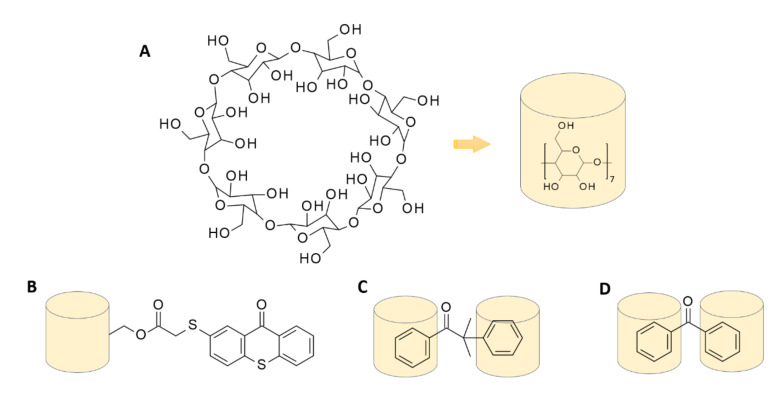
β-cyclodextrin structure and inclusion complexes of the host-guest type with photoinitiators; (**A**): β-cyclodextrin, (**B**): thioxanthone and β-cyclodextrin, (**C**): benzophenone and β-cyclodextrin, (**D**): 2,2-dimethoxy-2-phenyl acetophenone and β-cyclodextrin.

An interesting way to increase the solubility of hydrophobic initiators in water is through the host-guest chemical interaction. Cyclodextrins are cyclic oligosaccharides built from different amounts of optical active groups, called glucopyranose units (Figure 15 A) [230]. Due to their unique molecular structure, these compounds have the ability to cluster into small molecules with a hydrophobic cavity and hydrophilic outer surface and are, thus, able to form host-guest-type systems [231,232,233]. Many scientists have, therefore, proposed to increase the solubility of initiators by creating inclusion complexes with different types of cyclodextrin [234]. Balta et al. created thioxanthone photoinitiators using host-guest interactions with β-cyclodextrin (Figure 15 B) [235]. In turn, Temel et al. developed inclusion complexes using benzophenone and methylated β-cyclodextrin (Figure 15 C) [236]. Similar initiating systems for hydrogel formation were obtained by Ayub through complexation of 2,2-dimethoxy-2-phenyl acetophenone and methylated-β-cyclodextrin (Figure 15 D) [237]. Such a procedure ensured that a transparent hydrogel with a high degree of cross-linking was obtained. Xing et al. developed a 7-bis(2-(4-pentaneoxyphenyl)-vinyl)anthraquinone and a 2-hydroxypropyl-β-cyclodextrins (2-HP-β-CDs) initiating system, which allowed the production of hydrogel materials with the use of two-photon polymerization [238].

## 8. Multi-Component Water-Soluble Photo Initiating Systems

**Figure 16:**
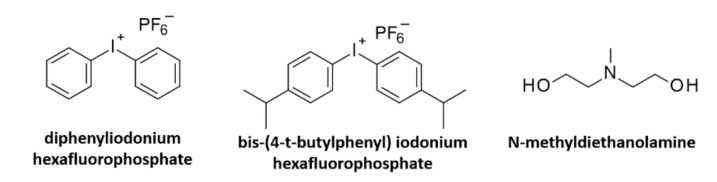
The structures of widely used additives in multi-component initiating systems for biomedical applications.

Multi-component initiating systems are very popular in the processes of photo induced polymerization reactions, mainly due to the fact that their absorption characteristics can be compatible with the emission characteristics of the light sources, such as UV-A-LEDs and Vis-LEDs. Such systems are also used in biomedical applications. Zuo et al. proposed to use of commercial fluorescent brighteners (styrene-based, coumarin-based, and 2,5-bis(benzoxazolyl)thiophene-based derivatives) together with commercially available iodine salt–diphenyliodonium hexafluorophosphate–to create hydrogel materials [239]. Coumarin derivatives together with water-soluble N-methyldiethanolamine acted as a multi-component initiating system to create a hydrogel with (hydroxyethyl)methacrylate [240]. The same amine co-initiator was used to obtain hydrogel through an amine–diketopyrrolopyrrole (DKPP) derivative system [241,242]. The structures of widely used additives in multi-component initiating systems for biomedical applications are shown in Figure 16.

## 9. Fields of Application for Water-Soluble Photoinitiators

In recent years, polymeric hydrogels have garnered plenty of interest in terms of their potential application due to the fact that their structural and biochemical properties are similar to those of the extracellular matrix (ECM) of most tissues [229]. Moreover, they show high porosity, which ensures high permeability to nutrients, oxygen, and metabolic products. The properties of these materials can also be adapted to the mechanical properties of soft tissues. Hydrogels for tissue engineering should be hydrophilic in order to promote cell adhesion, while the three-dimensional structure of these scaffolds should be porous to facilitate cell and nutrient diffusion [184,243]. Hydrogels are produced by cross-linking hydrophilic monomers or oligomers. Although hydrogels can be formed by conventional polymerization methods, e.g., thermally, using thermal initiators or initiators acting on the principle of redox reaction, polymerization under the influence of light is of the greatest interest. Compared to other methods, photopolymerization has many advantages: it is a very fast reaction (lasting from a few seconds to a few minutes) and allows spatial control over the resulting hydrogel, which permits the creation of various shapes that fit into the tissue structure. Currently, photo induced systems for the production of hydrogels include: radical polymerization under the influence of ultraviolet (UV) and visible (Vis) lights in water, or two-photon photopolymerization and thiol-en photopolymerization [245,246,247]. Hydrogels with an interpenetrating polymer network structure are also becoming increasingly more popular [248]. Photocured hydrogel materials are used in numerous applications, e.g., biosensing [249,250], encapsulation [18,251,252], drug delivery systems [253,254], scaffolding for the cell culture [255,256], in situ polymerization [257,258], and even direct polymerization in living cells [259,260]. All techniques of 3D printing are highly developed [261,262], including laser writing [263,264], inkjet bioprinting [265], and stereolithography [202,266,267]. Other applications include the production of various materials, including scaffolds [268] and layered hydrogels using surface photopolymerization [269].

## 10. Conclusions

In conclusion, interest in water-soluble photoinitiators has been ongoing for almost half a century. Significant developments in medicine, including nanomedicine [270], promote the advancement of photopolymerization processes, as well as the necessary initiating systems in the near future. The currently available modern technologies of nanomedicine, such as targeted drug therapy, modern analysis, and diagnostics of diseases, and the production of materials for cell or tissue culture, will require new and increasingly improved initiators that will meet all the criteria for the introduction of materials into the medical market. 

The development of water-soluble initiating systems is likely to take two directions. First, it will be based on the synthesis of completely new Type I or Type II photoinitiators with a wide absorption range reaching the visible range and, additionally, fulfilling a number of other requirements, such as lack of cytotoxicity, biocompatibility, and high initiation efficiency. Such photoinitiators can be applied, among others, in the processes of in situ polymerization, in targeted drug delivery, and in cell encapsulation, which may positively affect the treatment of some diseases, such as type I diabetes by the encapsulation of islets of Langerhans. 

The second direction of development is the study of two-photon photoinitiators (2PP), which will allow the effective production of hydrogel materials containing living cells with the use of 3D laser printing with extremely high resolution. The constant challenge is to obtain initiators with a simple and inexpensive synthesis path in which the scale can be easily transferred to the industry. 

This literature review has presented previous achievements in the field of water-soluble initiators in biomedical applications and has pointed at likely development paths and potential applications of photopolymerization processes.

## Figures and Tables

**Table 1:** Comparison of spectroscopic properties, solubility, and toxicity of water-soluble monoacylphosphine oxide (MAPO) and bisacylphosphine oxide (BAPO) derivatives [142].

Initiator	Derivative of	Spectroscopic Properties		Solubility [g/dm^3^]	Toxicity LC_50_ [mmol/dm^3^]
		λ_max-ab_ [nm]	ε _@λmax-ab_ [dm^3^·mol^-1^·cm^-1^]		
LAP	MAPO	380.5	191	47	3.1
TPO-Na	MAPO	380.5	250	29	< 0.56
BAPO-OLi	BAPO	383.5	197	54	2.6
Bapo-ONa	BAPO	383.5	256	60	2.8

LC_50_ – determines cytotoxicity in the cell culture. LC_50_ corresponds to the concentration of a given medium, which is fatal for 50% of cells.

**Table 2:** Summary of the main water-soluble initiators used in biomedical applications, their basic properties, and photo induced cleavage of photoinitiators.

Type of Initiator	Name of Initiator	Structure, Together with a Simplified Scheme of Photoinduced Cleavage of Photoinitiator	Maximum Absorbance / Source of Irradiation	Key Strengths	Key Drawbacks	Ref.
Type I	**Irgacure 2959**	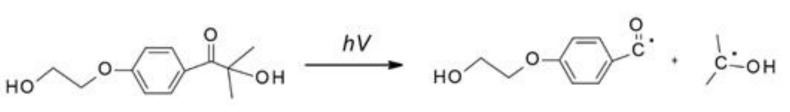	276 nm/ 365 nm	High initiation rate, low cytotoxicity, and immune- genicity	Low initiation efficiency, need for UV light sources, low water solubility (<5 w.%)	[104]
Type I	**TPO**	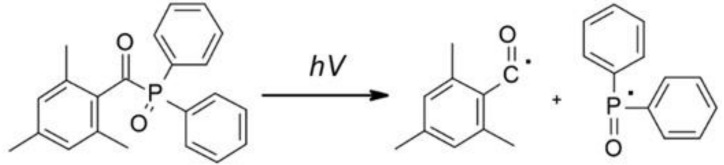	267, 298, and 380 nm	Cleaves into highly reactive radicals, good thermal stability	Poor water solubility	[146]
Type I	**LAP**	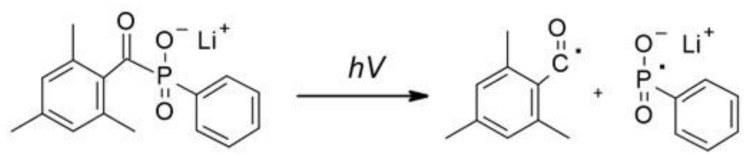	375 nm/ (320-390 nm) 405 nm	Good water solubility, possibility of using UV and visible light sources	Low initiation efficiency, especially when exposed to light from the visible range	[138], [142]
Type I	**BAPO-OLi**	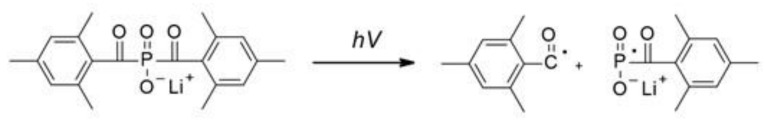	375 nm/ (320-420 nm)	Good water solubility ~54 g/l	Very low extinction coefficient in the range above 400 nm	[142,143,144]
Type I	**VA-086**	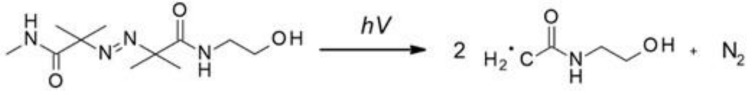	365 nm/ (365-385 nm)	Low toxicity, providing 90% cell survival, high initiation rate, good water solubility	Released nitrogen causes bubble formation	[97], [150,151,152]
Type II	**Eosin-Y**	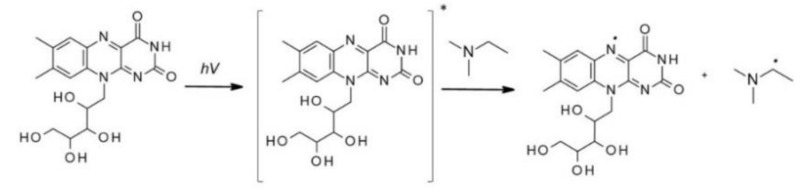	528 nm/ (400-800 nm)	Good water solubility, low cytotoxicity, wide range of absorbance, possibility to use different light sources in visible range, possibility to use low light powers	A second ingredient is needed for high initiation efficiency – the co-initiator	[158], [159,243]
Type II	**Camphor-** **quinone**	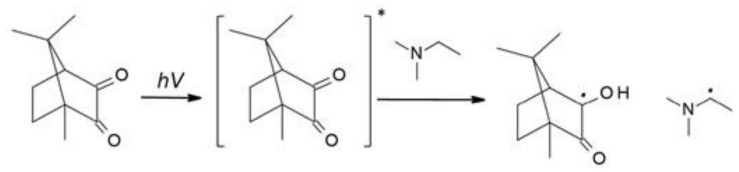	444 nm/ (400 -500 nm)	Wide absorption range based on the visible range	Modification needed to increase solubility in water, strongly yellow after reaction	[105], [186]
Type II	**Riboflavin**	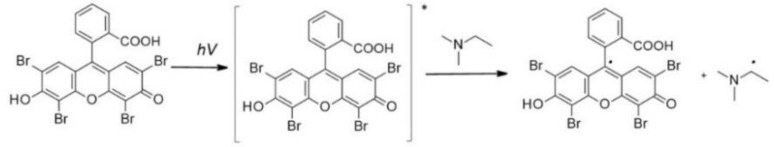	223, 267, 373 and 444 nm / (300-500 nm)	Excellent water solubility, wide absorption range, also in the visible area, non-toxic, beneficial to cells	Possibility of creating reactive oxygen species	[167], [177]
2PP	**WSPI**	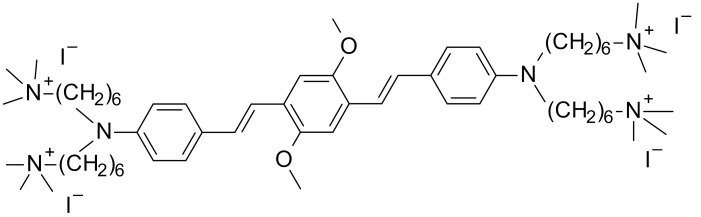	source of irradiation: laser – 800 nm	very good water solubility, excellent optical sensitivity, and resolution, no toxicity	significant limitations of speed fabrication	[207] [221] [244]
2PP	**BDEA**	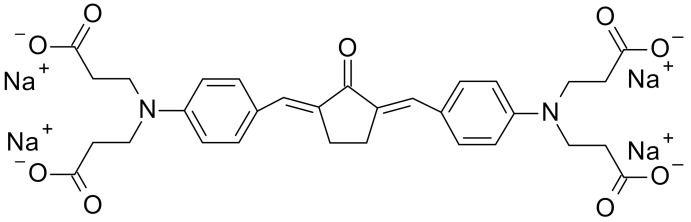				[224]
2PP	**P2CK**	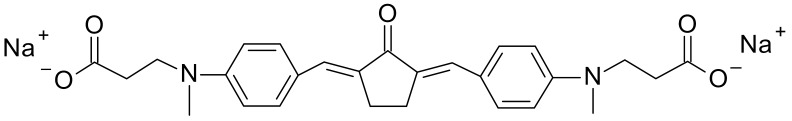				[225]
